# A rare case of primary carcinoma of axillary accessory breast tissue

**DOI:** 10.1093/jscr/rjab473

**Published:** 2021-10-20

**Authors:** Oishi Sikdar, Meghna Roy, Zaid Al-Ishaq, Veena Shinde, Tapan Sircar

**Affiliations:** Imperial College School of Medicine, London, UK; University of Birmingham, Birmingham, UK; Department of General Surgery, Royal Wolverhampton Hospitals NHS Trust, Wolverhampton, UK; Department of Pathology, Royal Wolverhampton Hospitals NHS Trust, Wolverhampton, UK; Department of General Surgery, Royal Wolverhampton Hospitals NHS Trust, Wolverhampton, UK

## Abstract

Carcinomas of primary accessory breast tissue are rare, comprising 0.3–0.6% of all breast cancers and occur most commonly in the axilla. We report the unusual case of a 50-year-old lady with mucinous adenocarcinoma of axillary accessory breast tissue. In this report we review the presentation, key investigations and treatment of this condition.

## INTRODUCTION

Accessory breast tissue is found in 2%–6% of the general population and is less common in Caucasians [1]. It is formed due to an abnormality in breast development but results in similar pathologies to pectoral breast tissue. Primary accessory breast carcinomas are rare, comprising 0.3%–0.6% of all breast cancers [2] and occur most commonly in the axilla [1]. Here we report a rare case of primary axillary breast carcinoma in the UK.

## CASE HISTORY

A 50-year-old, previously fit and well perimenopausal Asian lady presented with a 3-month history of an enlarging lump in the right axilla. She had no family history of breast or ovarian cancer. On clinical examination, both breasts were normal, she had accessory breast tissue on both sides. Within the right accessory breast area was a dominant 2 cm discrete indeterminate subcutaneous lump but no axillary lymphadenopathy.

**Figure 1:**
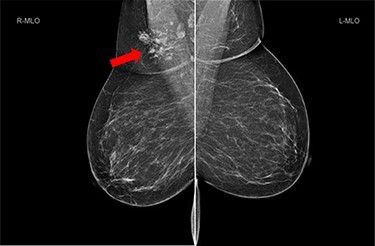
Bilateral mammogram shows mixed density glandular breast tissue with no focal abnormality within the breast. The right accessory breast tissue had increased in size and density (red arrow) from her previous mammogram with distortion and indeterminate imaging appearance, M3 (Royal College of Radiologist, Breast Group Classification).

**Figure 2:**
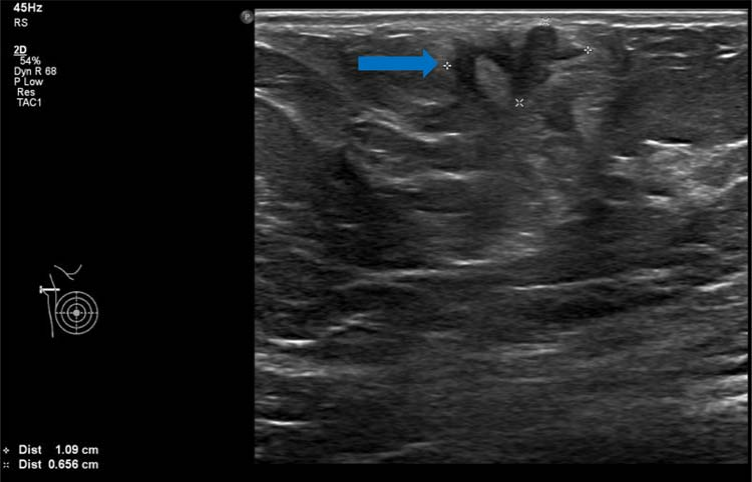
Ultrasound scan of right axillary accessory breast tissue shows focal area of illdefined hypoechogenicity underlying the dermis measuring 10.9 × 8.3 mm (blue arrow). No associated significant hypervascularity. The imaging appearance are indeterminate, U3 (Royal College of Radiologist, Breast Group Classification).

**Figure 3:**
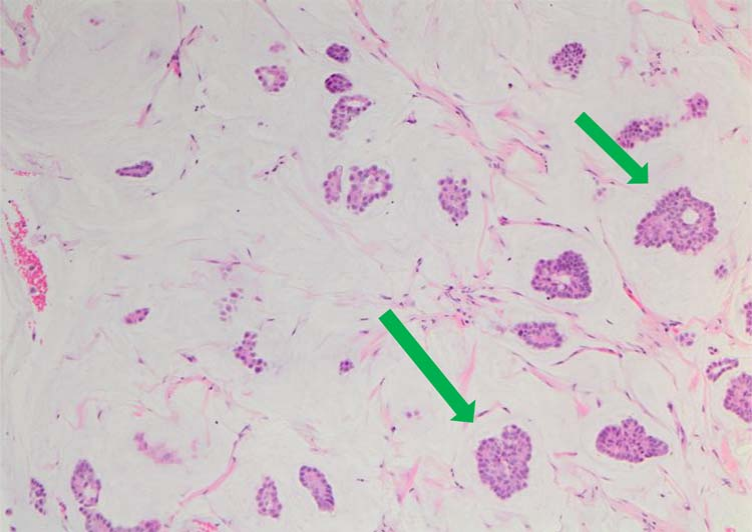
Nests of tumour cells with intermediate-grade nuclei are seen within pools of extra-cellular mucin 10× haematoxylin and eosin stain (green arrow).

She underwent triple assessment. Bilateral mammogram showed no abnormality in either breast. However, right axillary accessory breast tissue had notably increased in size since her previous mammogram (7 months earlier) and displayed slight distortion (R3) (Fig. 1). Ultrasound scan of the right axilla showed accessory breast tissue measuring 56 × 50 mm in its maximum dimension without any abnormal axillary lymph nodes. Furthermore, the palpable lump showed indeterminate characteristics (U3) measuring 10.9 × 8.3 mm (Fig. 2). Ultrasound-guided core biopsy of the abnormality showed Grade 1 mucinous adenocarcinoma (Fig. 3) which was oestrogen and progesterone receptor positive (Quick score of 8) and Her-2 negative. There was no evidence of lymphoid tissue in the core biopsy.

Following discussion in the multidisciplinary meeting the patient underwent total excision of right axillary accessory breast tissue and sentinel lymph node biopsy. Post-operative histology confirmed a 52 mm, Grade 2 mucinous adenocarcinoma with high grade ductal carcinoma *in situ*, oestrogen receptor positive and Her-2 negative. Two sentinel lymph nodes biopsied were both negative. However, the patient required a second surgery to achieve clear margins.

**Figure 4:**
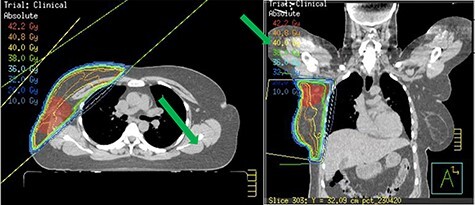
Radiotherapy plans showing boost to tumour bed.

Staging bone and CT scan showed no evidence of distant metastasis. She underwent Oncotype DX test for risk stratification and consideration of chemotherapy. The Oncotype DX recurrence score was 15 with group average absolute chemotherapy benefit <1%. Following careful consideration and discussion, chemotherapy was avoided. The patient received 4 weeks of forward-planned IMRT adjuvant radiotherapy to the right breast along with boost to the tumour bed (Fig. 4). She was commenced on tamoxifen due to her perimenopausal status.

## DISCUSSION

During embryogenesis, the mammary ridges, or ‘milk lines’, extend from the anterior axillary folds to the inguinal folds [3]. Incomplete regression of these ridges leads to development of accessory breast tissue along the milk lines. Development of this tissue is hormone dependent and usually becomes apparent during puberty and pregnancy [4]. Accessory mammary tissue in the axilla behaves like anatomical breast, their physiology and pathologies are similar and include pain, inflammation, fibroadenoma and carcinoma [4]. Other differential diagnoses for an axillary mass include axillary lymphadenopathy, lipoma, abscess, seroma, hidradenitis and skin lesions [4]. The incidence of malignancy in accessory breast tissue is around 0.3–0.6% [2]. The most common malignant pathology seen in accessory breast tissue is invasive ductal carcinoma [3] but there are reports of lobular cancer, medullary cancer and phylloides tumour [4]. Our patient had primary mucinous carcinoma, not to be confused with mucinous micropapillary carcinoma, which accounts for <0.2% of all breast cancer cases [5].

Literature suggests, accessory breast tissue cancers can have poor outcomes [4]. This may be attributed to delay in diagnosis due to rarity of the condition, delayed presentation, unclear investigative pathways and routine mammography often may not include axillary accessory breast tissue within the image field. Therefore, it is imperative that axillary breast tissue is assessed by triple assessment with careful clinical examination, mammogram and ultrasound scan and biopsy when indicated. Additionally, patients with accessory breast tissue should be made aware of the importance of monitoring this tissue for changes in size, consistency or any new lump.

One report suggests magnetic resonance imaging (MRI) scan of the breast to exclude occult breast cancer [2]. We feel this should be considered if biopsy of the lump within the accessory breast tissue showed features of lymphoid tissue thus suggesting axillary lymph node metastasis rather than a primary breast cancer. In our patient the ultrasound scan of axilla showed normal axillary nodes and the core biopsy showed invasive breast cancer with no lymphoid tissue. MRI should also be considered in patients with very dense anatomical breast where mammogram may not be totally diagnostic.

Controversy exists regarding surgery. Earlier reports recommended ipsilateral mastectomy whereas more recent reports suggest there is no additional advantage with mastectomy over total excision of accessory breast tissue [4, 6]. Total excision of accessory breast tissue including cancer and sentinel node biopsy is recommended. Axillary clearance is performed if preoperative lymph node biopsy is positive. Mastectomy is not required if there is no lesion noted within the anatomical breast on triple assessment. Our patient had no abnormality within the anatomical breast on examination and mammogram. Further adjuvant treatment namely chemotherapy, radiotherapy and endocrine treatment depends on the histology result and oncotype DX score. In our patient, Oncotype DX results did not show significant benefit of chemotherapy, hence this was avoided. As she was young and had T3 tumour we offered her adjuvant radiotherapy along with boost to the axillary tumour bed to reduce local recurrence. She was also recommended endocrine treatment.

## CONCLUSION

Clinicians should be aware of the possibility of primary breast carcinoma within axillary accessory breast tissues. If there is a dominant nodule within accessory breast tissue, then triple assessment is mandatory. Total excision of accessory breast tissue including the cancer area and sentinel lymph node biopsy is recommended if there is no other malignant lesion within the ipsilateral anatomical breast. Axillary clearance is performed only if axillary nodes are involved. Adjuvant treatment will depend on the tumour characteristics. Patients with accessory breast tissue should be informed to report to their doctor if they notice any changes in size or consistency or a new lump within the accessory breast tissue.
